# Effects of multienzyme supplementation on energy and nutrient digestibility in various feed ingredients for pregnant gilts

**DOI:** 10.1093/jas/skaf017

**Published:** 2025-01-29

**Authors:** Garrin L Shipman, Jorge Y Perez-Palencia, Jinsu Hong, Yanxing Niu, Anna Rogiewicz, Rob Patterson, Crystal L Levesque

**Affiliations:** Department of Animal Science, South Dakota State University, Brookings, SD 57007, USA; Department of Animal Science, South Dakota State University, Brookings, SD 57007, USA; Department of Animal Science, South Dakota State University, Brookings, SD 57007, USA; Department of Animal Science, University of Manitoba, Winnipeg, MB, Canada R3T 2N2; Department of Animal Science, University of Manitoba, Winnipeg, MB, Canada R3T 2N2; CBS Bio-Platforms Inc., Calgary, AB, Canada T2C 0J7; Department of Animal Science, South Dakota State University, Brookings, SD 57007, USA

**Keywords:** cereal grains, energy, fiber, gestation, multienzyme, protein feeds

## Abstract

The utilization of exogenous fiber-degrading enzymes in commercial swine diets is a strategy to increase the nutrient and energy density of poorly digestible ingredients. In a prior set of studies, dietary multienzyme blend **(ME**_**blend**_) supplementation increased the apparent total tract digestibility (**ATTD**) of nutrients, non-starch polysaccharides, and energy in complete high-fibrous gestation diets by 6% when fed to gestating sows. The current study aimed to determine the effects of ME_blend_ (containing xylanase, β-glucanase, cellulase, amylase, protease, pectinase, and invertase activities) supplementation on ATTD of energy and nutrients of individual feedstuffs commonly used in gestating sow diets across major pork-producing regions worldwide, which differ in their fibrous components. Twenty-seven gilts (initial body weight 176 ± 6.6 kg), in a crossover design with 4 periods (periods 1, 2, 3, and 4 from days 41 to 55, 56 to 70, 71 to 85, and 86 to 100 of gestation, respectively), were allocated to one of 7 diets (with or without ME_blend_ supplementation at 0.1% inclusion; 7 to 8 observations per treatment) to determine the ATTD of energy and neutral detergent fiber. Three diets contained corn, wheat, and sorghum as the sole source of energy. In the other diets, soybean meal (**SBM**), field peas (**FP**), canola meal (**CM**), and sugar beet pulp (**SBP**) each replaced 25% of the corn in the corn diet to determine the energy value of individual feedstuffs. Data were analyzed using a Student’s *t*-test to evaluate the effect of enzyme supplementation on these feedstuffs. The ME_blend_ increased the metabolizable and net energy of corn (*P *= 0.10) and wheat (*P *< 0.01) by 2% and 3%, respectively. The energy content of sorghum was not impacted by ME_blend_. Furthermore, a 6%, 4%, and 25% increase was observed in metabolizable and net energy of SBM, FP, and CM, respectively (*P *≤ 0.05). The energy value in SBP was not affected by ME_blend_ supplementation. In conclusion, supplementing diets with a multienzyme blend increased the energy content of corn, wheat, soybean meal, FP, and CM fed to gestating sows by approximately 2% to 25%, depending on the feedstuffs. The energy value of sorghum and SBP was not affected by the multienzyme blend. This should be considered when formulating fibrous diets for gestating sows to increase nutrient utilization of feedstuffs.

## Teaser

When fed to pregnant gilts, a multienzyme blend improved the apparent total tract digestibility of fiber and dietary energy content in corn, wheat, soybean meal, field peas, and canola meal by approximately 2% to 10%, depending on the feedstuffs. The effect of the multienzyme blend was more significant in protein feedstuffs than cereal grains and limited for sorghum and sugar beet pulp.

## Introduction

Reproducing sows consume approximately 20% of the total feed used in production systems (Ball and Moehn, 2013). When formulating least-cost rations, the regional availability of feedstuffs is considered, resulting in variation in feed ingredient usage across the main pork-producing regions worldwide, including Canada, China, the United States, the European Union, and Brazil (Bikker and Jansman, 2023). In the U.S. corn belt, adequate growing conditions make corn the economically favored cereal grain providing dietary energy in swine diets. On the other hand, in regions with shorter crop-growing seasons, such as parts of Canada or Northern Europe, wheat is more prevalent. Furthermore, in regions with a greater likelihood of drought conditions, sorghum is often the cereal grain of choice (Sauber and Owens, 2001). Protein sources also vary regionally, with canola meal (**CM**) being widely used in Western Canada and Europe due to its local production and oil processing capacity. Meanwhile, the global increase in soybean cultivation for oil extraction resulted in soybean meal (**SBM**) being the primary protein source in many regions. Field peas (**FP**) with their high starch and protein content, provide caloric and protein density to diets (Woyengo and Zijlstra, 2021). Fiber sources like sugar beet pulp (**SBP**) are valued in gestation diets because fiber sources increase the bulk density of feed, which stimulates stretch receptors in the gastrointestinal tract and signals satiety in limit-fed gestating sows (Bach Knudsen and Jørgensen, 2001). These feedstuffs, however, vary in their dietary fiber and non-starch polysaccharides (**NSP**) content and profile, which can hinder nutrient digestibility and energy availability (Knudsen, 1997, 2014).

The use of exogenous NSP-degrading enzymes in commercial swine diets is a strategic approach to increase nutrient and energy availability of poorly digested ingredients, thereby reducing ingredient usage and lowering diet costs (Torres-Pitarch et al., 2019). Prior set of trials have demonstrated that supplementing high-fiber gestation diets with a multienzyme blend (**ME**_**blend**_) increased the apparent total tract digestibility (**ATTD**) of nutrients, NSP, and energy by 6% in gestating sows (Shipman et al., 2023). Research on the effects of multicarbohydrase has primarily been conducted on its impact on the digestibility of common feedstuffs in growing pigs, rather than complete diets (Torres-Pitarch et al., 2019). However, there is limited data on the effects of multicarbohydrase on nutrient digestibility in feedstuffs when included in gestation diets for swine. This study aims to address this gap by evaluating the effects of ME_blend_ supplementation on ATTD of energy and nutrients, as well as the energy content of feedstuffs commonly included in gestating swine diets in the major pork-producing regions worldwide. The evaluated feedstuffs differ in their chemical composition, including starch, protein, and fiber content.

## Materials and Methods

Experimental procedures were conducted at the South Dakota State University Swine Education and Research Facility in Brookings, SD, USA, following approval by the Institutional Animal Care and Use Committee (2310-003A).

### Feedstuffs and dietary treatments

All ingredients (Table 1) originated from sources in the upper Midwestern United States in spring 2023. A total of 7 test diets were formulated. Three diets were formulated to contain corn, wheat, and sorghum as the sole energy source (Table 2). For the other 4 diets, SBM, FP, CM, and SBP were added to the corn diet replacing 25% of the corn to calculate energy content using the difference procedure (Zhang and Adeola, 2017). Minerals and vitamins were added to meet or exceed the NRC (2012) recommendations for gestating sows. The ME_blend_ (Superzyme, CBS Bio-Platforms Inc., Calgary, AB, Canada) was included at 0.1% in each of the 7 test diets at the expense of the base cereal grain resulting in 14 dietary treatments. The ME_blend_ was prepared to target a range of NSP, starch, carbohydrates, and protein. Enzyme preparation supplied 3,000 U of xylanase, 500 U of β-glucanase, 2,000 U of cellulase, 12,000 U of amylase, 6,000 U of protease, 2,000 U of pectinase and 700 U of invertase per 1 kg of diet. Titanium dioxide was included at 0.3% of the diet as an indigestible marker to determine nutrient digestibility (Zhang and Adeola, 2017). Diets were fed in mash form, and water was provided ad libitum to animals.

**Table 1.** Analyzed chemical composition of feedstuffs (%, as-fed basis)

Item	Corn	Wheat	Sorghum	Soybean meal	Field peas	Canola meal	Sugar beet pulp
GE, kcal/kg	3,903	3,843	3,880	4,123	3,976	4,330	3,762
DM	88.0	89.0	88.1	88.5	88.4	90.0	91.7
CP (N × 6.25)	8.7	15.3	9.1	47.9	23.8	40.3	9.2
EE	3.4	2.3	3.1	2.0	1.7	3.1	1.4
Starch	64.8	66.8	64.0	0.6	43.5	3.5	0.9
Ash	1.1	1.8	1.8	5.8	2.6	6.3	7.3
NDF	8.4	12.9	15.6	8.6	15.7	27.0	37.6
ADF	2.6	4.2	7.1	5.7	7.0	20.6	22.4
Total NSP	6.6	8.8	8.5	14.1	13.1	17.8	55.0
Arabinose	1.1	2.1	1.3	2.0	2.3	3.8	17.4
Xylose	1.5	3.4	2.4	1.0	1.2	1.4	0.8
Mannose	ND	ND	ND	0.7	ND	ND	0.7
Galactose	0.3	0.0	0.0	4.2	0.7	1.4	3.7
Glucose	3.2	2.9	4.3	3.4	6.5	6.1	16.4
Uronic acids	0.6	0.5	0.6	2.9	2.5	5.1	16.0
Water-soluble NSP	1.5	1.5	1.0	1.8	1.6	0.8	5.8
Water-insoluble NSP	5.1	7.3	7.5	12.4	11.6	17.0	49.2
TDF	10.9	14.5	17.4	16.3	21.2	33.7	67.2

GE, gross energy, DM, dry matter; CP, crude protein; EE, ether extract, NDF, neutral detergent fiber; ADF, acid detergent fiber; NSP, non-starch polysaccharides; TDF, total dietary fiber; ND, not detected.

**Table 2.** Ingredient and analyzed chemical composition of the experimental diets (%, as-fed basis)^1^

Item	Corn	Wheat	Sorghum	Soybean meal	Field peas	Canola meal	Sugar beet pulp
Ingredient composition							
Corn	97.4	—	—	72.8	72.5	72.7	72.8
Wheat	—	97.8	—	—	—	—	—
Sorghum	—	—	97.5	—	—	—	—
Soybean meal	—	—	—	25.0	—	—	—
Field peas	—	—	—	—	25.0	—	—
Canola meal	—	—	—	—	—	25.0	—
Sugar beet pulp	—	—	—	—	—	—	25.0
Limestone	0.9	1.1	1.0	0.8	0.9	1.1	0.3
Dicalcium phosphate	1.1	0.5	0.9	0.9	1.0	0.8	1.4
Salt	0.2	0.2	0.1	—	0.2	-	0.2
Sow vitamin premix^2^	0.1	0.1	0.1	0.1	0.1	0.1	0.1
Sow trace mineral premix^3^	0.2	0.2	0.2	0.2	0.2	0.2	0.2
TiO_2_	0.3	0.3	0.3	0.3	0.3	0.3	0.3
Analyzed composition							
GE, kcal/kg	3,810	3,797	3,783	3,809	3,776	3,858	3,718
DM	87.2	88.5	87.7	88.3	88.5	88.2	89.6
CP (N × 6.25)	8.7	14.8	8.6	20.4	12.6	16.5	8.6
EE	3.1	1.6	3.0	2.7	2.4	2.9	2.2
Starch	63.0	65.3	62.6	47.2	57.9	48.0	47.4
Ash	3.7	3.4	3.4	4.1	3.6	4.5	4.9
NDF	8.3	12.0	11.8	8.5	12.0	14.0	16.2
ADF	2.4	3.7	5.5	3.8	4.0	6.9	7.5
Total NSP	7.1	7.8	7.3	8.5	9.3	8.9	18.9
Arabinose	1.1	1.7	1.1	1.3	1.4	1.6	4.4
Xylose	1.5	2.8	1.8	1.4	1.4	1.4	1.3
Mannose	ND	ND	ND	0.2	ND	ND	ND
Galactose	0.3	0.3	0.0	1.2	0.4	0.6	1.2
Glucose	3.8	2.6	4.1	3.1	5.1	3.6	6.5
Uronic acids	0.5	0.4	0.5	1.2	1.1	1.7	5.5
TDF	10.8	13.8	13.9	12.5	16.4	16.9	25.6

GE, gross energy, DM, dry matter; CP, crude protein; EE, ether extract, NDF, neutral detergent fiber; ADF, acid detergent fiber; NSP, non-starch polysaccharides; TDF, total dietary fiber; ND, not detected.

^1^Multienzyme blend was included at 0 and 0.1% at the expense of the base cereal grain to create 2 different dietary treatments.

^2^J & R Distributing Inc., 518 Main Ave, Lake Norden, SD 57248, USA. Minimum provided the following per kg of diet: calcium 55 mg, vitamin A 11,000 IU, vitamin D_3_ 1,650 IU, vitamin E 55 IU; vitamin B_12_ 0.044 mg, menadione 4.4 mg, biotin 0.165 mg, folic acid 1.1 mg, niacin 55 mg, d-pantothenic Acid 60.5 mg, vitamin B_16_ 3.3 mg, riboflavin 9.9 mg, thiamin 3.3 mg.

^3^J & R Distributing Inc., 518 Main Ave, Lake Norden, SD 57248, USA. Minimum provided the following per kilogram of diet: zinc 170 mg as zinc hydroxychloride and zinc polysaccharide complex, iron 80 mg as iron polysaccharide complex, manganese 40 mg as manganese hydroxychloride and manganese polysaccharide complex, copper 20 mg as copper chloride and copper polysaccharide complex, iodine 0.4 mg as ethylenediamine dihydroiodie, and selenium 0.3 mg as selenium yeast and sodium selenite.

### Animals, experimental design, and sample collection

Following confirmation of pregnancy, 27 gilts (Camborough L1050-PIC × Duroc; initial bodyweight 176 ± 6.6 kg) in a single group were allocated to one of the 14 dietary treatments in a crossover design with 4 periods (periods 1, 2, 3, and 4 from days 41 to 55, 56 to 70, 71 to 85, and 86 to 100 of gestation, respectively) to determine ATTD of energy and neutral detergent fiber (**NDF**). This allowed for a minimum of 7 observations for each dietary treatment, which were balanced within the period. Sows were housed individually in gestation stalls (0.68 m × 1.98 m) equipped with a nipple drinker and a dry feeder. Sows were limit-fed 2.2 kg/d of their assigned experimental diet in a single feeding (0800 hour). In each period, diets were fed for 14 d with days 0 to 9 considered an adaptation period (Choi et al., 2024) followed by 5 d of the total collection of urine and grab fecal samples (days 11 to 14).

Urinary catheters (Lubricath, 2-way; Bard Medical Division, Covington, GA, USA; 18 Fr × 30 mL) were inserted on day 9 of each period and connected to closed containers in the same manner described by Miller et al. (2016). Urine sample collection commenced on day 10 and continued until 3 successful 24-h collections of urine were achieved. Three-day collection period was deemed adequate for a representative sample (Miller et al., 2016) and to avoid potential contamination from urinary tract infections (Levesque, unpublished data). A 24-h flush period was allowed if a sow pulled out her urinary catheter and displayed signs of urinary tract infection before reinserting the catheter. Sulfuric acid (98% H_2_SO_4_; 10 to 20 mL/24 h) was added to the collection containers to stabilize pH; containers were weighed and 10% of each 24 h collected urine by weight was stored at −20 °C. Urine samples were thawed following the conclusion of the experiment to be pooled within the sow and collection period, subsampled, and stored at −20 °C until further analysis. Fecal samples were collected once daily by rectal palpation, pooled within the sow and collection period, and stored at −20 °C until analysis. Rectal palpation was sufficient to induce a complete defecation from which fecal samples were collected.

### Chemical analyses

Prior to analysis, fecal samples were freeze-dried (Dura-Dry, Fits Systems, Kinetics Thermal Systems, Pennsauken, NJ, USA). Samples of complete diets with or without ME_blend_ were pooled within feedstuff for chemical analysis. Feedstuffs, diets, and fecal samples were finely ground to pass through a 0.75-mm screen using a centrifugal mill (Ultra Centrifugal Mill ZM 200, Retsch, Haan, Germany). The dry matter (**DM**) content of the feedstuffs, diets, and fecal samples was determined by drying samples at 102 °C for 24 h using a drying oven (Myers et al., 2004). NDF was measured on the feedstuffs, diets, and fecal samples according to AOAC procedure 2002.04 by an Ankom fiber analyzer (ANKOM Technology, Macedon, NY, USA). Titanium concentration of the diet and fecal samples was determined by spectrophotometry (model SpectraMAX190, Molecular Devices, Sunnyvale, CA, USA) at 408 nm after ashing at 525 °C for 10 h and digesting with anhydrous sodium sulfate and sulfuric acid at 120 °C for 24 h (Myers et al., 2004).

Samples of the feedstuffs and diets were analyzed for crude protein (**CP**) (method 968.06), ether extract (method 920.39), and ash (method 923.03) according to AOAC (2005). Acid detergent fiber (**ADF**) in diets and feedstuffs was analyzed using an Ankom 200 Fiber Analyzer (ANKOM Technology) according to the method of van Soest et al. (1991). Total and water-insoluble NSP was determined by gas–liquid chromatography (component sugars) using SP-2340 column and Varian CP-3380 GC (Agilent Technology, Mississauga, Canada) and by colorimetry (uronic acids) using Biochrom Ultrospec 50 (Biochrom Ltd, Cambridge, UK) according to the procedure described by Englyst and Cummings (1984) with some modifications (Slominski and Campbell, 1990). In brief, a 100-mg sample was treated with dimethylsulphoxide and incubated overnight at 45 °C with a solution of starch-degrading enzymes composed of amylase and amyloglucosidase (Sigma, St. Louis, MO, USA). Ethanol was then added, the mixture was left for 1 h, centrifuged, and the supernatant was discarded. The dry residue was dissolved in 1 mL of 12M H_2_SO_4_ and incubated for 1 h at 35 °C. Six milliliters of water and 5 mL of myo-inositol (internal standard) solution were then added, and the mixture was boiled for 2 h. One mL of the hydrolysate was then taken and neutralized with 12 M ammonium hydroxide, reduced with sodium borohydride, and acetylated with acetic anhydride in the presence of 1-methylimidazole. The water-insoluble NSP was determined by modifying the procedure: after overnight incubation, the solubles were discarded following centrifugation. The water-soluble NSP content was calculated by the difference between the total and water-insoluble NSP.

Total dietary fiber (**TDF**) was determined by a combination of NDF and neutral detergent soluble NSP measurements to address the solubility of NSP in the neutral detergent solution that causes a loss of NSP on the NDF assay. TDF was calculated as the sum of NDF and ND-soluble NSP (Slominski et al., 1994). Neutral detergent soluble NSP were calculated as the total sample NSP minus NSP present in the NDF residue. Total starch content in the diets and feedstuffs was measured using an assay kit (K-TSTA-100A, Megazyme Total Starch assay kit; Megazyme International Ltd, Wicklow, Ireland) following the manufacturer’s procedures.

The feed, feces, and urine gross energy (**GE**) contents were analyzed using bomb calorimetry (Parr 6300 calorimeter, Parr Instruments Co., Moline, IL, USA). For fecal samples, 1 g of ground feces was pressed into a pellet using a pellet press (Parr Instruments Co.), and placed into the bomb calorimeter. The sample was analyzed in duplicate and repeated if the difference between the 2 values was more than 2%. Analysis of urine GE followed the procedure as described by Kim et al. (2009).

### Calculations

The ATTD of energy and nutrients was calculated according to the indirect evaluation method using the marker approach. The following formula was used to calculate the ATTD of nutrients:


$$\begin{array}{l} ATTD,\;\%\;=\;100\;\;\;\left[100\;\times \;\left(\frac{Ti_{d}\times \;C_{f}}{Ti_{f}\;\times \;C_{d}}\right)\right] \end{array}$$

where Ti_d_ = concentration of titanium in the diet; Ti_f_ = concentration of titanium in feces; C_f_ = concentration of the component in feces and C_d_ = concentration of the component in the diet (Zhang and Adeola, 2017).

Digestible energy (**DE**) and metabolizable energy (**ME**) of diets (kcal/kg of DM and as-fed) were calculated following the procedure of Adeola (2001):


$$\begin{array}{l} DE=\frac{GE\;intake-GE\;output}{GE\;intake} \end{array}$$

$$\begin{array}{l} ME=\frac{GE\;intake-GE\;output-GE\;urine}{GE\;intake} \end{array}$$

The metabolizability of GE (ME: GE, %) was calculated as ME/GE × 100.

Energy content (DE and ME) and digestibility of DM, NDF, and GE of SBM, FP, CM, and SBP were calculated using the difference procedure (Kong and Adeola, 2014):


$$\begin{array}{l} D_{ti}\;=\;\;\;\frac{\left[D_{td}\;-\;\left(D_{td}\;\times \;D_{bd}\right)\right]}{P_{ti}} \end{array}$$

in which *D*_bd_, *D*_td_, and *D*_ti_ are the digestibility (%) of the component in the basal diet, test diets, and test ingredient, respectively, and *P*_bd_ and *P*_ti_ are the proportional contribution of the component by the basal diet and test ingredient to the test diet, respectively.

The net energy (**NE**) values of the feedstuffs (kcal/kg of DM and as-fed) were also calculated from the ME and analyzed macronutrient content using prediction equations (1) to (7) from NRC (2012):


$$\begin{array}{lll} NE= & \left(0.726\times ME\right)+\left(1.33\times EE\right)+\left(0.39\times starch\right)\; & \left(0.62\times CP\right)\left(0.83\times ADF\right) \end{array}$$

where all nutrient and digestible nutrient contents are expressed as gram per kilogram DM, the calculated NE on a DM basis was also recalculated to be expressed on an as-fed basis.

### Statistical analysis

The UNIVARIATE procedure of SAS (Version 9.4, SAS Inst. Inc., Cary, NC) was used to confirm the homogeneity of variance and to analyze for outliers. Data were analyzed using the MIXED procedure of SAS. Supplementation of ME_blend_ (with or without) was the fixed effect, and the collection period was used as the random effect. A student’s *t*-test was determined to be the most suitable model to evaluate the effect of enzyme supplementation on feedstuffs. Tukey’s adjusted means test was used to detect differences among treatments where *P* ≤ 0.05 was considered significant. Statistical tendencies were also reported if 0.05 < *P *≤ 0.10. Values are presented as means and standard error of the mean.

## Results

### General observations

One gilt aborted during the first period and was consequently removed from the study. Another gilt was found deceased during the 4th collection period. A veterinary pathologist determined that the causes of abortion and death were not related to the dietary treatments. Samples collected from these gilts during the periods of their respective incidents were discarded and not considered for further analysis.

### Chemical analysis of feedstuffs

Table 1 reports the chemical composition of the feedstuffs selected for evaluation in this study. As anticipated, the samples differed in protein and starch content, as well as in the TDF and its components. Among the cereal grains (corn, wheat, and sorghum) wheat had the highest starch and CP content. Sorghum contained more TDF, NDF, and ADF when compared to corn and wheat, but its NSP content was lower than that of wheat. FP is a source of both protein and starch, as they contain moderate amounts of both. Soybean meal and CM represent protein sources containing low starch but are different in the TDF and its components. Both CM and SBP contain a high amount of pectic polymers, as indicated by the relatively high concentrations of uronic acids and glucose. The presence of xylose in the feed ingredients indicates xylans and xyloglucan. TDF and water-insoluble NSP are greatest in SBP, followed by CM. The relative proportion of water-soluble of NSP to total NSP was higher in SBP than in CM, i.e., 10.5% vs 4.4% water-soluble NSP, respectively.

### ATTD in cereal grains

Tendencies were observed for ME_blend_ supplementation in increasing the metabolizable GE (ME:GE, %), ME, and predicted NE (in DM and as-fed basis) of corn with a *P*-value of 0.1 (Table 3). However, no other effects on corn were observed with ME_blend_ supplementation. Supplementing with the multienzyme blend increased (*P *< 0.01) the ATTD of DM, NDF, and GE in wheat, as well as the DE and ME content (both in DM and as-fed basis). The predicted NE of wheat increased with ME_blend_ supplementation (*P *< 0.01). In contrast, ME_blend_ supplementation had no effect on the nutrient digestibility and energy of sorghum.

**Table 3.** ATTD of nutrients and energy in cereal grains fed to gestating sows with (+) or without (−) multienzyme blend (ME_blend_)

Feedstuff	Corn		SEM	*P-*value	Wheat		SEM	*P-*value	Sorghum		SEM	*P-*value
ME_blend_	–	+			–	+			–	+		
*n*	7	7			8	8			7	7		
ATTD, %												
DM	86.7	86.7	0.73	0.958	88.3	91.1	0.54	<0.001	84.2	85.1	0.55	0.265
NDF	50.1	54.7	4.79	0.374	63.9	73.1	1.27	<0.001	61.3	62.9	2.13	0.600
GE	88.2	88.7	0.76	0.538	89.3	91.9	0.46	<0.001	84.1	85.8	0.66	0.117
DE, kcal/kg												
DM	3,794	3,813	33.0	0.538	3,810	3,921	18.2	<0.001	3,575	3,643	27.9	0.117
As-fed	3,338	3,355	29.0	0.538	3,392	3,491	16.2	<0.001	3,183	3,244	2.1	0.117
ME:GE, %	86.2	88.0	1.11	0.099	88.8	91.2	0.45	<0.001	83.5	84.7	0.86	0.374
ME, kcal/kg												
DM	3,708	3,787	47.6	0.099	3,786	3,888	19.1	<0.001	3,549	3,597	36.4	0.374
As-fed	3,262	3,331	33.0	0.099	3,371	3,462	17.0	<0.001	3,160	3,203	27.9	0.374
NE, kcal/kg^1^												
DM	2,945	3,002	34.6	0.099	2,930	3,004	13.9	<0.001	2,773	2,808	26.5	0.374
As-fed	2,590	2,641	30.4	0.099	2,609	2,675	12.3	<0.001	2,469	2,501	23.6	0.374

SEM, standard error of the mean; DM, dry matter; NDF, neutral detergent fiber, GE, gross energy; DE, digestible energy; ME, metabolizable energy; NE, net energy.

^1^NE (kcal/kg) = (0.726 x ME) + (1.33 x ether extract) + (0.39 x starch)—(0.62 × crude protein)—(0.83 × acid detergent fiber); (Equations (1) to (7); NRC, 2012).

### ATTD of protein and fiber sources

The ATTD of GE increased in the SBM and CM diets with ME_blend_ supplementation (*P *< 0.05) with no effects on the ATTD of DM and NDF (Table 4). DE (kcal/kg DM and as-fed) and ME:GE increased in the CM diets with ME_blend_ supplementation (*P *< 0.05). The ME:GE in the SBM diet tended to increase with ME_blend_ supplementation (*P *= 0.08). Multienzyme blend supplementation increased the ME (kcal/kg DM and as-fed) of the CM diet (*P *= 0.02) but tended to increase the ME content of the SBM diet (*P *= 0.08). Multienzyme blend supplementation had no impact on the digestibility or energy content of the FP and SBP diets.

**Table 4.** ATTD of nutrients and energy in test diets containing protein and fiber feedstuff fed to gestating sows with (+) or without (–) multienzyme blend (ME_blend_)

Feedstuff	SBM		SEM	*P-*value	FP		SEM	*P-*value	CM		SEM	*P-*value	SBP		SEM	*P-*value
ME_blend_	−	+			−	+			−	+			−	+		
*n*	8	8			7	7			8	8			7	7		
ATTD, %																
DM	86.3	86.9	0.76	0.159	87.4	87.9	0.75	0.884	78.5	79.7	1.11	0.345	80.7	80.6	0.54	0.902
NDF	53.2	54.0	3.27	0.772	70.1	72.2	2.60	0.503	47.4	50.3	3.65	0.555	65.6	63.3	3.28	0.442
GE	87.2	88.3	0.65	0.015	88.0	88.6	0.61	0.416	80.4	83.7	1.08	0.008	83.1	83.3	0.48	0.793
DE, kcal/kg																
DM	3,842	3,893	28.4	0.015	3,757	3,782	26.0	0.416	3,578	3,704	47.8	0.008	3,505	3,513	20.1	0.793
As-fed	3,391	3,436	25.1	0.015	3,325	3,348	23.0	0.416	3,154	3,265	42.2	0.008	3,139	3,147	18.0	0.793
ME:GE, %	86.1	87.0	0.78	0.075	87.4	87.8	0.61	0.341	79.8	82.9	1.23	0.018	82.4	82.5	0.62	0.944
ME, kcal/kg																
DM	3,795	3,834	33.0	0.075	3,727	3,745	25.8	0.341	3,531	3,669	54.4	0.018	3,477	3,480	26.2	0.944
As-fed	3,350	3,385	29.1	0.075	3,299	3,315	22.8	0.341	3,113	3,234	48.0	0.018	3,115	3,117	23.5	0.944

SBM, soybean meal; FP, field peas; CM, canola meal; SBP, sugar beet pulp; DM, dry matter; NDF, neutral detergent fiber; GE, gross energy; DE, digestible energy; ME, metabolizable energy.

The ATTD of DM and GE increased in SBM with ME_blend_ supplementation (*P *≤ 0.05; Table 5). The DE, ME, and predicted NE (kcal/kg DM and as-fed) of SBM were also increased with ME_Blend_ (*P *≤ 0.05). The ME:GE, ME (kcal/kg DM and as-fed), and predicted NE (kcal/kg DM and as-fed) in FP tended to increase with ME_blend_ supplementation (*P *= 0.09), though no other effects were observed for FP. The ME_blend_ increased the ATTD of GE, DE (kcal/kg DM and as-fed), ME (kcal/kg DM and as-fed), and predicted NE (kcal/kg DM and as-fed) of CM (*P *= 0.01). The nutritive value of SBP was unaffected by ME_blend_ supplementation.

**Table 5.** ATTD of nutrients and energy in protein and fiber feedstuff fed to gestating sows with (+) or without (−) multienzyme blend (ME_blend_)

Feedstuff	SBM		SEM	*P-*value	FP		SEM	*P-*value	CM		SEM	*P-*value	SBP		SEM	*P-*value
ME_blend_	−	+			−	+			−	+			−	+		
*n*	8	8			7	7			8	8			7	7		
ATTD, %																
DM	87.8	92.4	2.58	0.015	90.2	91.9	2.01	0.358	53.0	57.8	4.46	0.347	62.0	62.3	2.35	0.889
NDF	51.8	52.6	10.83	0.931	76.3	78.1	4.58	0.788	50.6	54.3	7.74	0.730	73.7	79.8	6.80	0.270
GE	83.4	86.9	2.58	0.050	86.9	88.2	2.44	0.655	58.1	68.4	4.41	0.014	66.7	67.0	1.90	0.906
DE, kcal/kg																
DM	3,980	4,138	113.5	0.049	3,685	3,740	104.1	0.656	2,860	3,308	187.4	0.014	2,735	2,742	81.9	0.951
As-fed	3,521	3,660	100.4	0.049	3,256	3,305	92.0	0.656	2,574	2,977	168.6	0.014	2,508	2,514	75.1	0.951
ME:GE, %	81.4	86.2	2.82	0.020	79.3	82.3	2.30	0.093	55.7	68.2	4.43	0.011	64.2	65.8	2.37	0.677
ME, kcal/kg																
DM	3,794	4,016	131.6	0.020	3,567	3,704	103.4	0.093	2,678	3,280	213.2	0.011	2,635	2,698	97.2	0.677
As-fed	3,356	3,552	116.4	0.020	3,152	3,273	91.4	0.093	2,410	2,952	191.9	0.011	2,417	2,474	89.1	0.677
NE, kcal/kg^1^																
DM	2,398	2,559	95.5	0.020	2,574	2,673	75.1	0.093	1,537	1,974	154.8	0.011	1,672	1,717	70.6	0.677
As-fed	2,121	2,263	84.5	0.020	2,275	2,363	66.4	0.093	1,384	1,777	139.3	0.011	1,533	1,575	64.7	0.677

SBM, soybean meal; FP, field peas; CM, canola meal; SBP, sugar beet pulp; DM, dry matter; NDF, neutral detergent fiber; GE, gross energy; DE, digestible energy; ME, metabolizable energy; NE, net energy.

^1^NE (kcal/kg) = (0.726 × ME) + (1.33 × ether extract) + (0.39 × starch) − (0.62 × crude protein) − (0.83 × acid detergent fiber); (Equations (1) to (7); NRC, 2012).

## Discussion

Exogenous feed enzymes are effectively utilized in modern livestock production to increase nutrient digestibility and feed efficiency. They are particularly effective at improving the nutritive value of poor-quality, high-fiber, or processed feed ingredients. Considering that adult gestating sows can digest energy more efficiently from feedstuffs compared to growing pigs (Lowell et al., 2015), ME_blend_ supplementation increased the ATTD of nutrients, NSP, and energy in high-fiber gestation diets by 6% when fed to gestating sows (Shipman et al., 2023). The effects of multienzyme preparations on the digestibility of individual common feedstuffs, rather than complete diets, have been evaluated in grower pigs (Torres-Pitarch et al., 2019). The results show the diversity of the NSP profiles of each feedstuff, which demonstrates the broad spectrum of potential substrates for enzymes. Thus, the objective of this current study was to evaluate the effect of ME_blend_ supplementation on the energy digestibility of various feedstuffs representative of global swine-producing areas when fed to gestating sows.

ME (both DM and as-fed) for the corn, wheat, and sorghum without ME_blend_ supplementation were similar to values for gestating sows reported by Lowell et al. (2015). Related to this, Wang et al. (2022) recently reported that the DE and ME of SBM fed to gestating sows were 4,271 and 4,039 kcal/kg, respectively, on a DM basis. It should be noted that the DE and ME observed in the study by Wang et al. (2022) are 10% greater than the DE and ME for SBM without ME_blend_ supplementation in the current study. The lesser energy values for SBM observed in the current study might be due to the use of young pregnant gilts, while Wang et al. (2022) conducted their trial with older pregnant sows. Regardless of feed intake, the energy value of feed can be 10% greater in older adult sows than in younger gilts (Casas and Stein, 2017).

The low NSP and fiber content in corn, wheat, and sorghum allow these cereal grains to be primary energy sources in swine diets, but the arabinoxylan content found in these cereal grains can reduce potential energy availability due to the insoluble fraction which can reduce gastric transit time and limit nutrient digestibility in the gastrointestinal tract (Jaworski et al., 2015; Gutierrez et al., 2014; Petry et al., 2020). At the same time, the soluble fractions found in arabinoxylan and β-glucan of cereal grains can increase the viscosity and thickness of digesta during transit in the gastrointestinal tract (Bach Knudsen, 2014). An increase in the viscosity of digesta can be problematic as endogenous digestive enzymes are unable to penetrate the viscous and sticky substance resulting in a reduction in the digestion and absorption of nutrients (Hooda et al., 2010).

In the current study, the supplementation of an ME_blend_ increased the ME and NE of corn by 2%, respectively, and produced a 3% increase in the ME and DE of wheat when fed to pregnant gilts. However, when compared to grower pigs, supplementation of single-component xylanase into high corn–fiber diets increased the DE and ME by 5% and 4%, respectively (Petry et al., 2020). It should be noted that xylanase hydrolyzes the β-1,4-arabinoxylan-glycosidic bonds of arabinoxylan which cleaves the fiber complexes and dietary arabinoxylan components while reducing the insoluble components in the small intestine (Petry et al., 2019). Solubility might be increased from the cleavage of the insoluble arabinoxylans by xylanase and result in delayed gastric emptying to allow for improved digestive and absorptive efficiency (Davidson and McDonald, 1998; Abelilla and Stein, 2019; Petry et al., 2024). This mode of action also occurs when xylanase is supplemented into wheat-based diets for grower pigs (Lærke et al., 2015). Emiola et al. (2009) observed that supplementing an ME_blend_ containing xylanase, β-glucanase, and cellulase activities increased the ATTD of energy, but not NDF, in corn–wheat dried-distiller grains with solubles-based diet fed to grower pigs. This aligns with the current study’s observation that the energy content of the corn was improved with ME_blend_ supplementation, without affecting the ATTD of NDF or DM.

While reduction in viscosity is consistent with carbohydrase supplementation in poultry (Raza et al., 2019), viscosity is not influenced by the supplementation of xylanase in corn-based diets as corn-based diets are relatively nonviscous and predominantly insoluble (Navarro et al., 2018; Petry et al., 2024). Pectic polymers also contain viscous and soluble properties that hinder nutrient digestibility (Jha et al., 2019). Generally, high molecular weight soluble fiber causes high viscosity in the stomach and small intestine with increasing retention time thereby disturbing the enzyme contact with nutrients inducing low digestibility of nutrients in the small intestine of monogastric animals (Jha et al., 2019). Serena et al. (2008) reported the viscosity of ileal digesta increased for gestating sows consuming low fiber and high insoluble fiber diets containing SBP. When considering SBP has 3 to 4 times greater soluble NSP and water-binding capacity than cereal grains (Navarro et al., 2018), there is a possibility that the activity of the exogenous ME_blend_ was hindered by the formation of gelatinous viscous digesta.

Interestingly, the effect of ME_blend_ on energy increase in wheat was 1% greater than that observed in corn. No studies have been published that directly compare the effects of ME_blend_ in corn versus wheat. However, Abelilla and Stein (2019), found that supplementing a single-component xylanase in wheat-based diets fed to growing pigs effectively increased the ATTD of GE, DE, and ME by 2%, while no such impact was observed in corn-based diets. Xylanase supplementation also increased the ATTD of water-insoluble fiber in wheat-based diets but had no effect on the water-insoluble fraction in corn-based diets. In the aforementioned study, the authors speculated the lack of a response by the specific xylanase used in their experiment might be due to its limitation in hydrolyzing the glycosidic and ester bonds in the arabinoxylans in corn, as the structure of arabinoxylan in corn differs from that in wheat (Vehmaanperӓ, 2022). This follows, given that the structure and functionality of xylanases and other carbohydrases can vary depending on the microbial organism the enzymes are produced (Vehmaanperӓ, 2022). Furthermore, the efficacy of enzymes is in part related to the balance between substrate availability and enzyme concentration (Olukosi and Adeola, 2013). Given that wheat contains higher concentrations of xylose and arabinose than corn, the greater substrate availability in the wheat may make the exogenous xylanase in ME_blend_ more effective compared to corn. Wheat also has a substantially more soluble fraction that increases transit time and allows for greater digestibility compared to corn, making it plausible that the ME_blend_ had an extended period of time to hydrolyze NSP while in the gastrointestinal tract (Keys and DeBarthe, 1974). Studies conducted using in vitro digestive modeling indicate that the ATTD of the NSP component sugars in wheat is 20% greater than in corn (Jaworski et al., 2015). This greater ATTD of the NSP component sugars and slower transit time in wheat combined with ME_blend_ supplementation, likely explains the higher energy increase observed in wheat.

The effects of enzyme supplementation in sorghum-based diets have received less attention because sorghum contains less soluble NSP than wheat and corn, and the viscosity is likely less relevant in this grain (Knudsen, 2014). In this study, 11.5% of NSP in sorghum is water-soluble NSP while 17.3% and 23% in wheat and corn, respectively. As the majority of NSP in sorghum was insoluble they can decrease transit time through the gastrointestinal tract and hinder enzyme activity through various mechanisms (Wenk, 2001). If insoluble NSP reduce gastric transit time, the ME_blend_ might not have had sufficient time to act on its substrates effectively. Tannins in sorghum are capable of inhibiting the activity of endogenous protease, lipase, and amylase activity thereby reducing energy utilization in pigs (Pan et al., 2021, 2023). The enzyme inhibitory nature of tannins appears to also be applicable to microbial fibrolytic enzymes, for example, lignocellulose hydrolysis is reduced when cellulase is complexed to tannin (Olsen et al., 2011).

Studies evaluating NSP-degrading enzymes in sorghum-based pig diets have been inconsistent, primarily because most used various combinations or enzyme cocktails, making it difficult to isolate the beneficial effects, if any. Park et al. (2003) found that including amylase and cellulase in finisher pig diets containing sorghum and SBM tended to increase average daily weight gain but did not affect feed efficiency, nitrogen digestibility, or carcass characteristics. Xylanase supplementation over a 42-d period improved the performance of animals fed sorghum-based diets (González-Ortiz et al., 2020). Neither growth performance nor feed efficiency were improved when growing pigs were fed sorghum–SBM-based diets supplemented with a xylanase and β-glucanase mixture (Oliveira et al., 2022). A 3% increase in DE or ME was observed by Oliveira et al. (2022) with the xylanase and β-glucanase mixture when fibrous ingredients (e.g., corn dried-distiller grains or wheat middlings) were included in sorghum-SBM diets fed to growing pigs but produced no effects when fibrous feedstuffs were not included. The results of the current and abovementioned studies suggest fibrolytic enzymes might not act on substrates originating from sorghum but rather on other feedstuffs mixed into sorghum-based diets. A limitation of exogenous NSP-degrading enzymes in complete diets is that the effects of enzymes on raw materials might not be additive or applicable to mixed feeds (Bedford, 2018).

Most enzyme sources used in animal feeds contain more than a single enzyme activity (Bedford and Classen, 1993) and most microorganisms used for enzyme production can produce multiple activities (Bhat and Hazlewood, 2001). For example, an organism isolated for xylanase activity has the potential to produce side activities, such as a mannanase, however, the secondary activity can be difficult to quantify relative to the primary activity (Bhat and Hazelwood, 2001). As all NSP are present in plant feedstuffs to varying degrees, an ME_blend_ containing a wider range of the NSP-degrading enzymes should enable greater hydrolysis, cleavage, and digestibility of NSP substrates compared to single-component carbohydrases. In an in vitro degradation trial, increasing the complexity of an enzyme blend degraded on average 25% more of the total NSP in the cell walls of wheat, CM, and SBM in comparison to incubating a single-component enzyme (Meng et al., 2005). Aligned with this concept, the supplementation of a more complex enzyme blend in diets fed to roosters was reported to have increased digestibility of the NSP sugar components but also improved degradation of starch granules and reduced digesta viscosity leading to better animal performances (Meng et al., 2005).

The effect of ME_blend_ was more pronounced in protein sources (e.g., SBM, FP, and CM) than in energy sources such as wheat and corn. Legumes, oil seeds, and their co-products contain a higher concentration of cellulose compared to cereal grains (Bach Knudsen, 1997). Due to structural similarities between cellulose and β-glucan, cellulases, and β-glucanases work conjunctively to hydrolyze and cleave either polymer into smaller oligosaccharides (Sadhu and Maiti, 2013). The enhanced nutrient digestion and energy increase in the protein sources most likely result from the combined activities of cellulase and β-glucanase supplied in the ME_blend_. While there was a protease included in the ME_blend_, the effect of the ME_blend_ on protein digestion cannot be differentiated although digestion of protein may have contributed to the reported increase in energy digestibility (Peng et al., 2024).

Legumes, canola, and their co-products (e.g., SBM and CM) contain substantial amounts of oligosaccharides such as stachyose, raffinose, and verbascose. The incomplete digestion of oligosaccharides in the small intestine can lead to enteric dysbiosis, resulting in poor nutrient digestion and reduced growth performance in monogastrics (Baker et al., 2014; Adewole et al., 2016). The mode of action of invertase is to catalyze the hydrolysis of d-glucose from d-fructose at the α-1,4-glycosidic bonds in α-d-galactosides, which are comprised of the raffinose family of oligosaccharides such as raffinose, verbascose, and stachyose (Manoochehri et al., 2020). Inclusion of invertase in combination with other carbohydrases has been researched in SBM diets to reduce the antinutritional effects of raffinose, stachyose, and verbascose while increasing DE and ME of the diet (Lee et al., 2019; Aranda-Aguirre et al., 2021). The ATTD of GE was increased by 10% for weaned pigs fed a wheat-CM-based diet when supplemented with a multienzyme preparation containing invertase (Omogbenigun et al., 2004). The fermentative capacity increases as pigs mature and may limit the response of invertase as the older animal is capable of digesting and fermenting raffinose (Longland et al., 1994). Greater substrate availability of the oligosaccharides present in SBM and CM can explain the greater increase in the protein feedstuffs as efficacy of enzymes is in part related to a balance between substrate availability and enzyme concentration (Olukosi and Adeola, 2013).

Soybean meal, a rich protein source with a highly digestible AA compared to other protein sources, showed a 6% increase in energy content when supplemented SBM-based growing pig diet with a multienzyme preparation comprising of cellulase, pectinase, mannanase, galactanase, xylanase, β-glucanase, amylase, and protease, as reported by Velayudhan et al. (2015). This is consistent with the 6% energy increase that was observed in SBM fed to pregnant gilts supplemented with ME_blend_ in the current study. Soybean meal contains a significant concentration of pectin polymers, as indicated by NSP uronic acids and galactose contents, as well as trace amounts of β-mannan. The greater increase in ATTD and energy in the SBM might have resulted from cellulases having the ability to degrade cellulose-mannan and cellulose-galactan structures, and pectinase can degrade cellulose-pectin and manno-pectin complexes (Sadhu and Maiti, 2013; Shrestha et al., 2023).

The ME and NE in FP increased by 4% with ME_blend_ supplementation. FP contain high concentrations of starch, along with moderate amounts of amino acids and fiber. They also have a high ratio of amylose to amylopectin in their starch granules (Woyengo and Zijlistra, 2021). Multienzyme preparation supplementation in barley–wheat-based diets containing FP had modest effects on the ATTD of DM and GE when fed to nursery pigs with no effects on overall growth performance (Thacker and Racz, 2001) However, a multienzyme preparation consisting of β-glucanase, phytase, protease, amylase, cellulase, and pectinase reduced the excretion of P and N in growing pigs fed diets containing FP (Nyachoti et al., 2006). Meng and Slominski (2005) found that the apparent ME of a corn–pea diet increased by 140 kcal/kg (as-fed basis) when a multienzyme preparation comprising of xylanase, β-glucanase, pectinase, cellulase, mannanase, and galactanase was included. Amylose, linked by α-1,4-glycosidic bonds, and amylopectin which is linked by α-1,6-glycosidic bonds, must be broken down by amylase or amyloglucosidase into smaller oligosaccharides and eventually individual glucose monomers for effective digestion (Cowieson, 2005). Supplementation with exogenous amylase can further break down amylose and amylopectin which escape endogenous amylase activity, thereby increasing the energy and starch digestibility of feedstuffs (Rupolo et al., 2023).

CM has lower protein but 3-fold the fiber concentration compared to SBM (Liu et al., 2016; Woyengo and Zijlstra, 2021). Consequently, the inclusion of CM in gestation diets must be limited compared to SBM when formulating dietary energy (Liu et al., 2018). Using a porcine in vitro digestion model, it was observed that including a multienzyme preparation increased the in vitro digestibility of NSP component sugars in CM (Lee et al., 2018). Reducing the NSP content and disrupting the fiber encapsulation improve energy availability and fermentability of the feedstuffs, contributing to increased energy. The 10% increase in the energy of the CM, when fed to gestating sows with ME_blend_ supplementation, is likely due to enzymatic action breaking fiber encapsulation. Previous studies on ME_blend_ supplementation in CM or diets containing CM in adult pregnant sows have shown mixed results. Velayudhan et al. (2018) found no effect of multienzyme preparation inclusion on the ATTD of GE in lactation diets containing CM. Similarly, Velayudhan et al. (2019) reported no effect of a multienzyme preparation (comprised of cellulase, pectinase, mannanase, galactanase, xylanase, β-glucanase, amylase, protease, and phytase) at 0.1% inclusion on the ileal digestibility of amino acids or CP when CM was fed to sows in mid- or late-gestation.

Multienzyme supplementation did not influence the ATTD or energy content of the SBP. Crome et al. (2023) reported that a multienzyme supplemented in a complete corn–SBM diets containing 20% SBP increased the ATTD of GE, DM, and NDF by approximately 5% in gestating sows. Additionally, a 4% increase in the DE and ME was observed in the corn–SBM–SBP diet with multienzyme supplementation, which included pectinase activity (Crome et al., 2023). Pectic polysaccharides, which include rhamnogalacturonan with associated side chains consisting of arabinose, galactose, and xylose residues, are the primary NSP in SBP (Abou-Elseoud et al., 2021). Exogenous pectinase can cleave the galacturonic polymer and de-esterify the methyl esters of the pectic backbone (Sharma et al., 2013). As stated previously, the effect of ME_blend_ might have been hindered by the greater water-binding capacity in the SBP.

In summary, including a multienzyme preparation in diets can increase ATTD of nutrients and energy for gestating sows fed corn, wheat, soybean meal, FP, and CM by approximately 2% to 25%, depending on the feedstuffs. Furthermore, the multienzyme blend had a greater impact on protein feedstuffs than on cereal grains but did not affect the energy value or digestibility of sorghum and SBP. From a practical standpoint, the exogenous multienzyme blend can enhance the energy content of feedstuffs for gestating sows while potentially reducing diet costs but may be less effective with sorghum or SBP.

## Abbreviations

ADF: acid detergent fiber
ATTD: apparent total tract digestibility
CM: canola meal
CP: crude protein
DE: digestible energy
DM: dry matter
EE: ether extract
FP: field peas
GE: gross energy
ME_blend_: multienzyme blend
ME: metabolizable energy
NDF: neutral detergent fiber
NE: net energy
NSP: non-starch polysaccharides
SBM: soybean meal
SBP: sugar beet pulp
TDF: total dietary fiber
